# Should fine needle aspiration biopsy be the first pathological investigation in the diagnosis of a bone lesion? An algorithmic approach with review of literature

**DOI:** 10.1186/1742-6413-4-9

**Published:** 2007-04-17

**Authors:** Ravi Mehrotra, Mamta Singh, Premala A Singh, Rahul Mannan, Vinod K Ojha, Pradumyn Singh

**Affiliations:** 1 Department of Pathology, Moti Lal Nehru Medical College, University of Allahabad, Allahabad, India

## Abstract

**Background:**

Fine needle aspiration biopsy (FNAB) is gaining increasing popularity in the diagnosis of musculoskeletal lesions; and in many patients, a definitive diagnosis can be rendered from aspiration smears alone. Its applicability in bone pathology, however, has been controversial due to a high percentage of inadequate smears, difficulty in evaluation of tissue architecture and nonspecific results in the diagnosis of primary bone lesions. In this study, the value of aspiration as the first pathological investigation in the diagnosis of a bone lesion was evaluated.

**Methods:**

91 cases of clinically suspected cases of bone lesions were aspirated over a period of two years. Direct or cytospin smears were fixed in 95% alcohol and stained by Hematoxylin and Eosin or air-dried and later fixed in methanol for May Grŭnwald Giemsa staining.

**Results:**

Of the 91 patients who were subjected to FNAB, 81 were considered satisfactory and 10.9 % (10) were inadequate\inconclusive for diagnosis. Cyto-histological concordance was obtained in 78.5 % (51/65) patients. Positive and negative predictive values were 87.5% and 97.2 % respectively. Sensitivity as a preliminary diagnostic technique was 93.3%, whereas specificity was 94.5 %. Overall, diagnostic accuracy was 94.2 %. Metastatic lesions were detected with 100% accuracy. Two cases were reported as false positive and one case as false negative.

**Conclusion:**

Cytology provides valuable information to the clinician to make an informed decision regarding appropriate therapy. We conclude that time-consuming and costly investigations may be reduced by choosing FNAB as the initial pathological diagnostic method for skeletal lesions of unknown origin. The choice of radiological examinations, laboratory tests and surgical biopsies can be determined after the FNAB diagnosis.

## Background

Fine needle aspiration biopsy (FNAB) has established its role in the preliminary diagnosis and planning of therapy for lesions in organs like thyroid, breast, lymph nodes and even the skull [1]. It, however, plays a limited role in detection of bone lesions. This may be attributed to lack of experience of the cytological appearance of bone lesions, which in turn, is due to difficulty associated with aspiration [2].

For long, widespread usage of FNAB as a diagnostic tool has been impeded because of the requirement of considerable training and experience of the cytopathologist as well as limited clinical information, false negative and false positive diagnoses and the overlap of cytological features in benign and malignant lesions. Often diagnosis of benign lesions cannot be made with certainty. Precise classification of a tumor is difficult on the basis of FNAB alone and histopathological confirmation is frequently required. However, on the other hand, FNAB of bone lesions has its own advantages of being simple, safe, and inexpensive and a quick outpatient procedure. It can also be repeated at different sites in case of inadequate material being aspirated [3].

This study was undertaken to assess the accuracy of FNAB and histopathological correlation in the diagnosis of bone lesions. Special emphasis was given to understanding its limitations and diagnostic aberrations. Analysis of the discordant cases was done to determine the source of diagnostic errors. A review of literature was done to compare the results with those of previous workers.

## Materials and methods

This study was conducted by the department of Pathology in conjunction with the department of Orthopedics, Moti Lal Nehru Medical College, Allahabad, India at the tertiary referral center: the Swaroop Rani Nehru Hospital. 91 cases were referred for FNAB in clinically suspected bone lesions.

A detailed clinical workup, including radiological assessment (X-ray and/or CT scan), was done prior to FNAB. The site of aspiration was approached through the shortest distance with radiological guidance. Aspirates were obtained using disposable needles (22–24 G) attached to a disposable 10 ml plastic syringe. A Cameco-type syringe holder was used where necessary. Under aseptic conditions, the needle was introduced into the lesion and after maintaining a negative suction pressure, multiple quick oscillations in different directions were made till some material was seen in the hub of the needle. Then the needle was withdrawn after releasing the negative pressure gently. One to two more passes were made into the lesion from different sites to ensure adequate sampling.

The physical nature of the aspirate was noted as fluid, pus, blood, caseous material and tissue bits etc. to be processed accordingly. Contents of the needle were blown on clean glass slides and the smears were made immediately. A few smears were quickly fixed in 95% ethanol for Hematoxylin and Eosin staining while the remaining smears were air dried and fixed in methanol for May Grŭnwald Giemsa staining. If sufficient material was left after preparing the smears, cell blocks were also prepared. In addition to the above mentioned routine stains; cytochemical stains like reticulin (Gomori's), alkaline phosphatase, Periodic acid Schiff (PAS) with without diastase, mucicarmine and immunohistochemical (IHC) stains were employed to support the diagnosis wherever necessary.

Cytodiagnostic light microscopy was embarked upon; all the smears were meticulously interpreted by two experienced cytopathologists. Accordingly, the smears were categorized as "benign", "malignant", "suspicious" and "inadequate\inconclusive". There was no perfect or absolute morphological feature of cancer, which when present unequivocally, meant that the cell is cancerous or when absent means that there was no cancer: however certain features, when taken in their totality and keeping in view the clinico-radiological findings enabled the cytopathologist to divide the cytologic findings into benign, suspicious and malignant. The smears were categorized as "suspicious" when the specimen was hypocellular and a few neoplastic cells were present or the cytological features of malignancy could not be ruled out conclusively and "inadequate\inconclusive" category was assigned when the specimens were extremely paucicellular or blood mixed to an extent that all other elements were obscured. The criteria used for labeling the aspirates as benign and malignant are depicted in Table 1. Wherever possible, an attempt was made to render an exact cytological diagnosis.

**Table 1:** Criteria used in differentiating benign and malignant cytology.

	Benign	Malignant
Nuclear membrane	Smooth	Irregular
Chromatin	Even	General disarray
Nucleolus	Not prominent	Enlarged, irregular and sharply angled
Mitosis		Abnormal mitosis
Nucleo- Cytoplasmic ratio	Normal or low	High

For histopathological examination, tissues were embedded in paraffin blocks, sliced into 2–3 micron sections and stained with routine Hematoxylin and Eosin staining and examined in a double blind fashion by two pathologists. In case of discrepancy, the opinion of a third pathologist was taken and two concordant diagnoses were treated as final.

The findings of FNAB were correlated with the histopathological diagnosis. Taking histopathology as the "gold standard" the diagnostic indices were calculated in terms of true and false positive, true and false negative, sensitivity, specificity, predictive values and accuracy test to support our study design. Calculation of these values was based on cases interpreted as diagnostic on histology, excluding both the inadequate\inconclusive smears as well as suspicious category.

A step-wise approach to FNAB diagnosis of bony lesions is given as follows:

Step 1: Establish category of clinical presentation

A patient may present with a bony mass/es under the following clinical scenarios: (i) Routine medical check-up, (ii) Bony pain, swelling or discharging sinus (iii) Known malignant cases. Important relevant data include age of the patient and site of involvement. Radiologic correlation is mandatory.

Step 2: Establish category of radiologic findings

In many instances, a preoperative diagnosis can be achieved with a high degree of accuracy based on non-invasive imaging techniques and close clinical correlation. FNAB is useful in defining those lesions without characteristic imaging appearance. Lists of entities along with the cytologic and radiological findings are given as a working guide. [see Additional file 1]

Step 3: Establish nature of cytohistologic findings.

Usual cytological findings are summarized in the "additional table".

Step 4: Further confirm nature of cytohistologic findings

The initial cytologic assessment is crucial as it forms the basis upon which ancillary tests are ordered; the results of which should be interpreted in the larger context of the case. Special stains and IHC may be helpful. A whole battery of antibodies is available for the comparative immunohistochemical study of primary and metastatic bone tumors, specially utilizing cell block preparations. The two major diagnostic issues are (i) whether the cells are malignant or benign? (ii) what is the histogenesis of the malignant cells? For example osteosarcoma exhibit strong positivity for Vimentin, variable for Actin/Desmin and S-100, if chondroid differentiation is present.

Step 5: Establish final diagnosis based on multidisciplinary approach

Close clinicopathological correlation is mandatory for enhancing the yield of FNAB diagnoses and the reduction of indeterminate reports.

**Figure 1:**
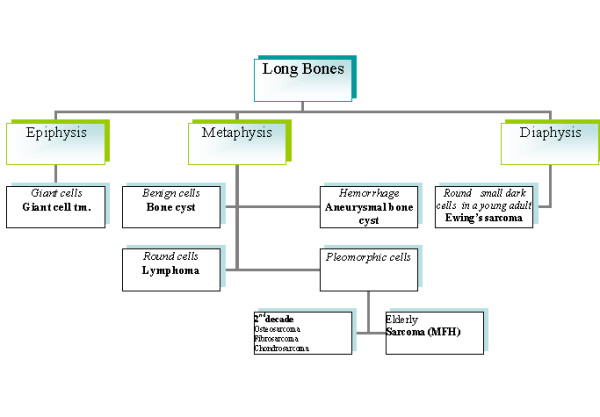
A diagnostic algorithm for FNAB diagnosis of long bone lesions spine in Fig. 2 and skull in Fig. 3.

**Figure 2:**
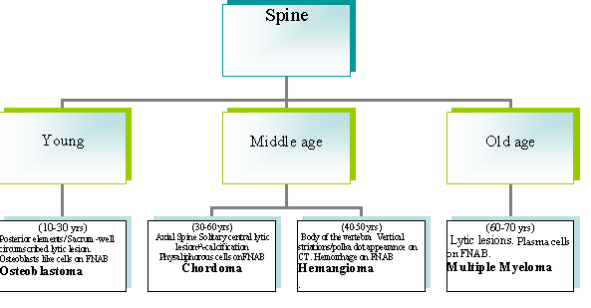
A diagnostic algorithm for FNAB diagnosis of long bone lesions spine in Fig. 2

**Figure 3:**
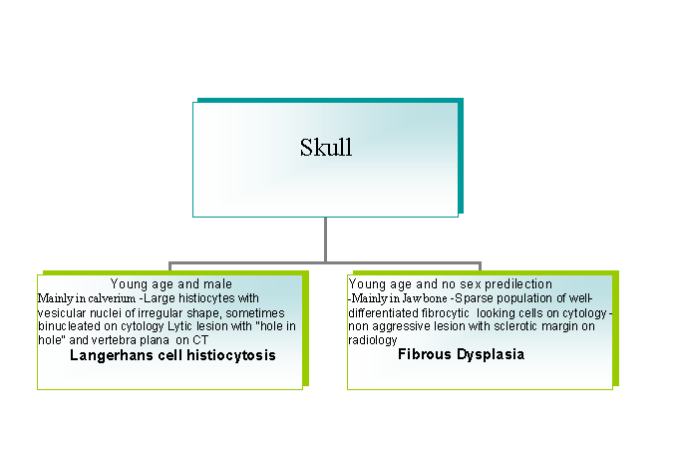
A diagnostic algorithm for FNAB diagnosis of long bone lesions skull.

A diagnostic algorithm for FNAB diagnosis of long bone lesions is given in Fig. 1, spine in Fig. 2 and skull in Fig. 3.

## Results

FNAB categories of 91 cases are summarized in Table 2. Of 91 cases, 81 (89%) were considered satisfactory and 10 (10.9%) were considered inadequate\inconclusive for diagnosis. The correlation of the diagnosis from FNAB and Histopathological examination (HPE) in 65 cases is shown in Table 3. Subsequent HPE was available for 65 (71.4%) of 91 cases, including 36 cases (76.6%) of 47 that were labeled cytologically benign, 3 (75%) of 4 that were labeled suspicious on cytology, 16 (53.3%) of 30 that were malignant on FNAB and all 10 cases in the inadequate\insufficient category. In the cytologically suspicious category, on histopathology, 2 cases (66.7%) out of 3 were found to be malignant. The sensitivity of FNAB was 93.3%, specificity 94.5%, positive predictive value 87.5% and negative predictive value 97.2%. The diagnostic accuracy was 94.23%. Of the 36 cases reported as benign on cytology, 1 proved to be malignant on histology, giving a false negative diagnosis. Of the 16 cases reported as malignant on cytology, 2 were later diagnosed as benign on histology, resulting in a false positive diagnosis.

**Table 2:** Cytohistological correlation in 65 bone lesions

Cytological Diagnosis	No. of cases with FNAB	No. of cases with histopathology	No. of benign cases	No of Malignant cases
Benign	47	36	35	01
Suspicious	4	03	01	02
Malignant	30	16	02	14
Inadequate\Insufficient	10	10	10	0
Total	91	65	48	17

**Table 3:** Cytological diagnoses in benign, suspicious, malignant, inadequate categories highlighting concordance.

			Cytological diagnosis			
Histology • BENIGN	No. of Cases	No. with 100% Cyto- histological concordance	Benign	Suspicious	Malignant	Inadequate

Non Ossifying Fibroma	2	-	-	-	-	2
Giant Cell Tumor	17	16	16	-	1	-
Osteomyelitis	3	3	3	-	-	-
Osteochondroma	4	2	2	2	-	-
Aneurysmal Bone Cyst	3	3	3	-	-	-
Osteoid Osteoma	2	-	-	-	-	2
Chondroma	2	2	2	-	-	-
Endostosis	1	1	1	-	-	-
Metaphyseal Fibrous Defect	1	-	-	-	-	1
Osteofibrous Dysplasia	2	-	-	-	-	2
Ameloblastic Fibroma	1	-	-	-	-	1
Giant Cell Reparative Granuloma	1	-	-	1	-	-
Cavernous Haemangioma	1	-	-	-	-	1
TB Osteomyelitis	3	2	2	-	1	-
Enchondroma	1	1	1	-	-	-
Normal Bone	1	-	-	-	-	1
Hemangioendothelioma	1	1	1	-	-	-
Hemangiopericytoma	1	1	1	-	-	-
Chondromyxoid Fibroma	2	2	2	-	-	-
• MALIGNANT						
Osteosarcoma	7	6	1	-	6	-
Ewing's Sarcoma	4	4	-	-	4	-
Metastatic Adenocarcinoma	1	1	-	-	1	-
Chondrosarcoma	2	2	-	-	2	-
Plasmacytoma	2	2	-	-	2	-
TOTAL	65	49	35	3	17	10

**Figure 4:**
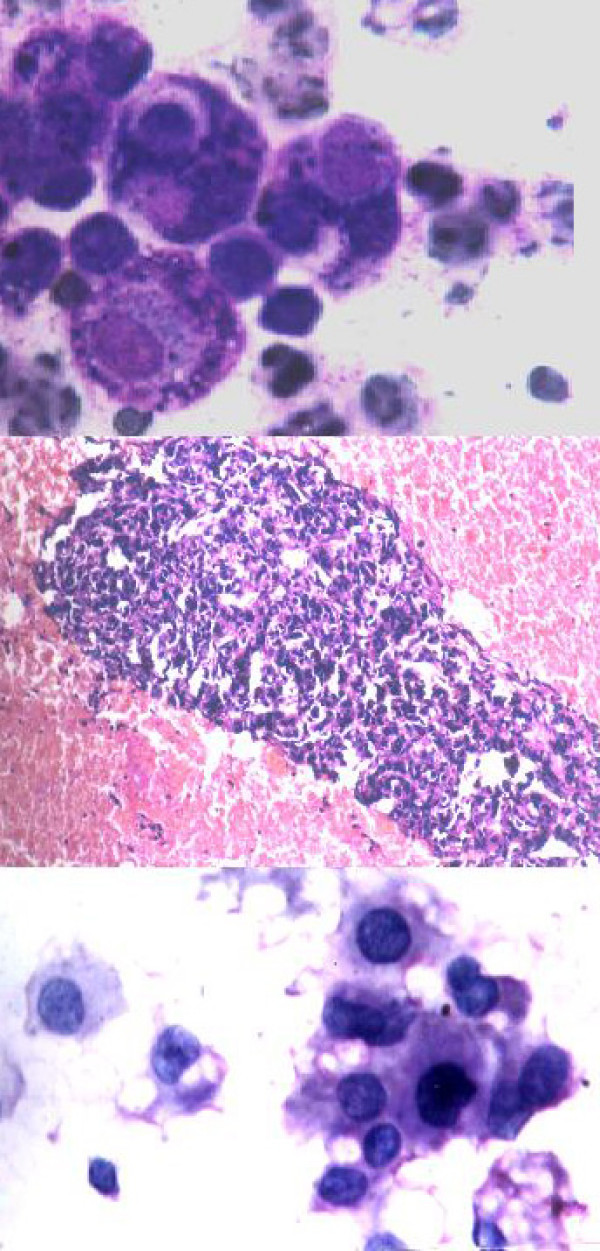
1. Cells showing coarse purplish granules in the cytoplasm [MGG; ×1000]. 2. Plasmacytoid tumor cells. [H&E; ×1000]. 3. Cell block preparation of osteosarcoma: [H&E; ×200].

**Figure 5:**
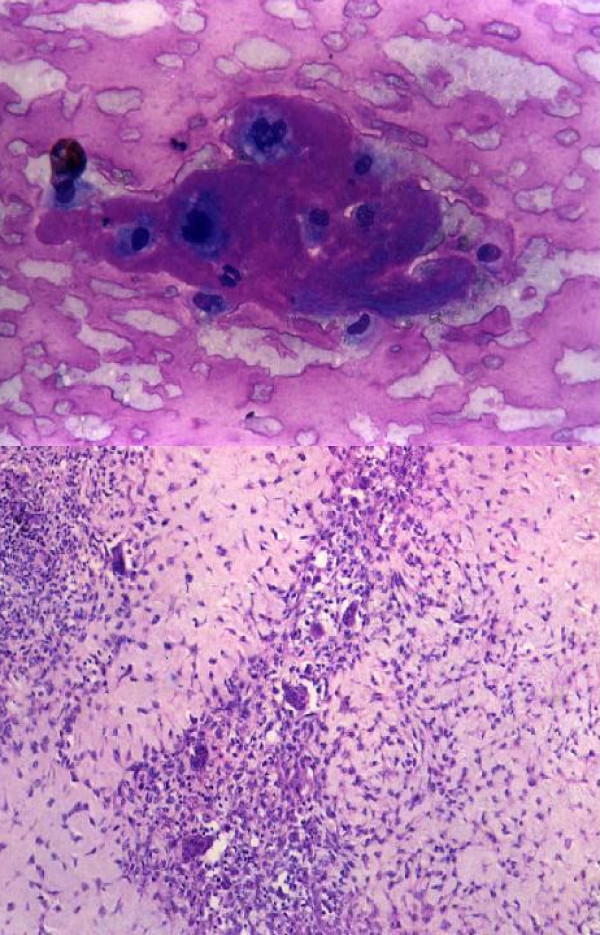
1. Chondroblast like cells embedded in chondroid fragment in a myxoid background. [MGG; ×400].

**Figure 6:**
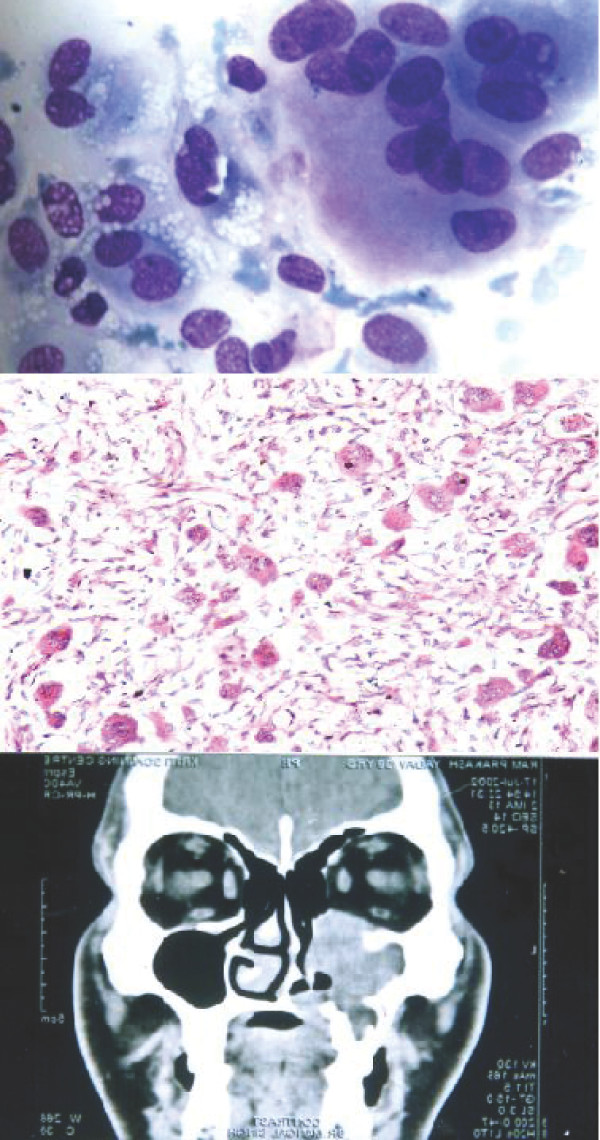
1. Giant cell tumor like picture of giant cell reparative granuloma [MGG; ×400]. 2. Giant cell reparative granuloma: histological picture showing giant cells lying in loose stroma. [H&E ×200]. 3. CT scan of giant cell reparative granuloma showing a mass in right maxillary sinus with extension into the adjacent areas.

**Figure 7:**
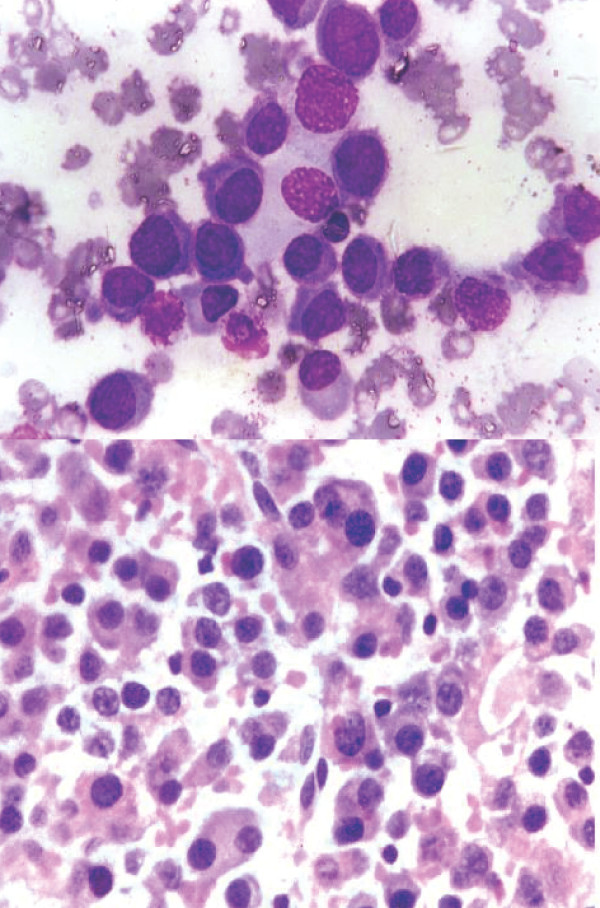
1. Smear showing myeloma cells. [MGG; × 400]. 2. Histopathology section showing dispersed tumor cells many having a "clock-face" condensation of chromatin. [H&E × 200].

**Figure 8:**
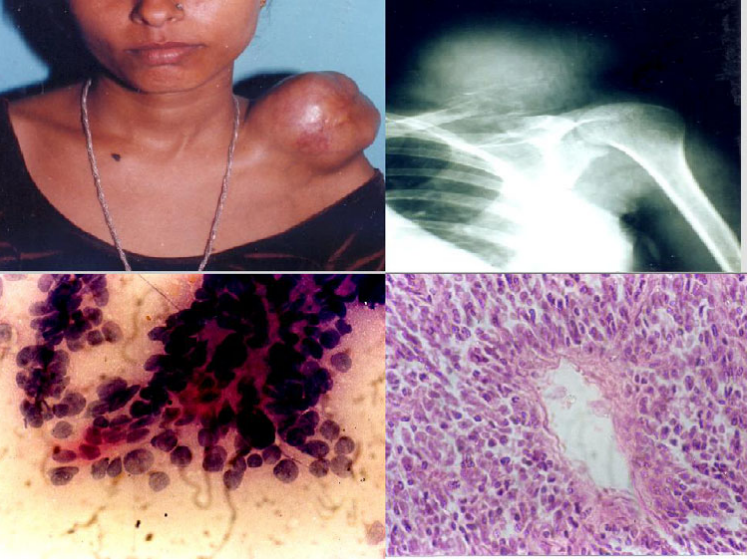
1. Photograph of a patient showing swelling over left shoulder: later diagnosed as Hemangiopericytoma. 2. X-ray showing lesion involving the left clavicle. 3. Smear showing malignant round cells radiating from vessels. [MGG ×400]. 4. Histological section of the same case showing monomorphic round cells radiating from cells [H&E × 200].

**Figure 9:**
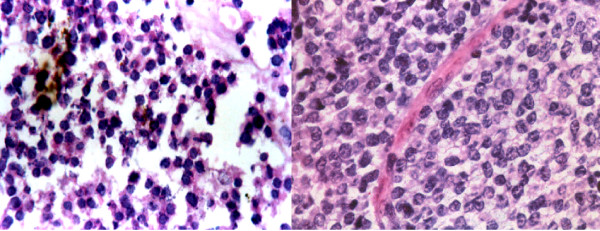
1. Cytology of Ewing's sarcoma. [MGG ×200]. 2. Histology of the same.

Table 4 shows cases correctly diagnosed on cytology. The cytodiagnosis included two cases of chondromyxoid fibroma (Fig. 4), haemangiopericytoma (Fig. 5), tuberculosis (Fig. 6), metastatic carcinoma (Fig. 7), Ewing's tumor (Fig. 8) and myeloma (Fig. 9).

**Table 4:** Cases in which specific cytological diagnosis was rendered.

Lesions • BENIGN	No. with specific cytological diagnosis was rendered.
Non Ossifying Fibroma	-
Giant Cell Tumor	16
Osteomyelitis	3
Osteochondroma	2
Aneurysmal Bone Cyst	3
Osteoid Osteoma	-
Chondroma	2
Endostosis	1
Metaphyseal Fibrous Defect	-
Osteofibrous Dysplasia	-
Ameloblastic Fibroma	-
Giant Cell Reparative Granuloma	-
Cavernous Haemangioma	-
TB Osteomyelitis	2
Enchondroma	1
Normal Bone	-
Hemangioendothelioma	1
Hemangiopericytoma	1
Chondromyxoid Fibroma	2
• MALIGNANT	
Osteosarcoma	6
Ewing's Sarcoma	4
Metastatic Adenocarcinoma	1
Chondrosarcoma	2
Plasmacytoma	2
TOTAL	49

**Figure 10:**
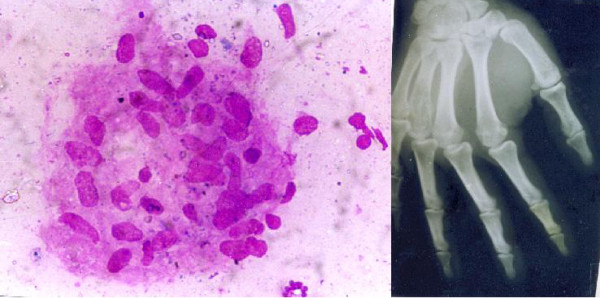
1. Epithelioid granuloma of tubercular osteomyelitis. [MGG ×400]. 2. X ray right hand from a case of tubercular osteomyelitis involving distal portion of 4^th ^metacarpal.

In 48 benign lesions, discordant diagnoses were made in 3 cases. They included 2 cases of chronic granulomatous bone disease which, on FNAB, appeared to be metastatic adenocarcinoma, the discrepancy was due to macrophages on cytology being misinterpreted for mucin rich cells. In retrospect, PAS staining was found to be negative in these cases and a diagnosis of metastatic adenocarcinoma was refuted. In addition, a case of giant cell reparative granuloma was wrongly diagnosed as giant cell tumor on cytology (Fig. 10).

**Figure 11:**
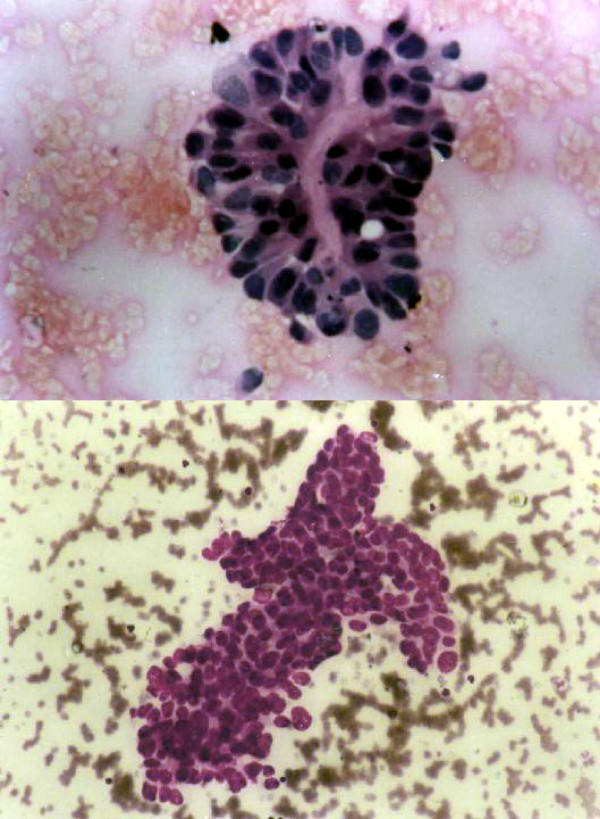
1. Metastatic adenocarcinoma: Tumors cell arranged in a glandular pattern [MGG ×400]. 2. Metastatic adenocarcinoma: Tumors cell arranged in an acinar pattern. [MGG X400].

Of the 17 malignant lesions, 14 were diagnosed as malignant, 2 as "suspicious" and 1 as benign on cytology. All the inadequate/inconclusive smears were found to be benign on histology. The single discordant diagnosis was seen in a case where a diagnosis of osteochondroma on cytology was found to be an osteosarcoma on histopathology. (Fig. 11)

## Discussion

Martin and Ellis first applied this technique to the diagnosis of bone lesions in 1930 [4]. Agarwal et al [5] and Layfield et al [6] have done pioneering work in describing the diagnostic accuracy and clinical utility of fine needle aspiration cytology in the diagnosis of clinically suspected primary bone tumors. As presurgical chemotherapy has become the standard treatment for osteosarcoma, FNAB has gained importance in recent years as an appealing diagnostic method [7].

Several published series have yielded overall accuracy values ranging from 51% to 100% (Table 5) [3-33]. This study differs from previous ones since it explores he utility of FNAB as the first pathological investigation in the diagnostic armamentarium of bony lesions. Of the 91 patients, histological confirmation was available for 65 patients. The sensitivity of FNAB was found to be 93.3%, specificity 94.5%, positive predictive value 87.5% and negative predictive value 97.2%. Diagnostic accuracy, on the other hand, was 94.23%. Similarly, Agarwal et al [5] reported the FNAB findings in 226 cases of bone tumors. A specific morphologic diagnosis on FNAB was possible in 159 cases with one false positive and 29 false negative reports. Giant cell tumor (32%) and Ewing's sarcoma (22%) were the most common bone tumors encountered. In their series, the overall sensitivity and specificity was 86% and 94.7% respectively. The positive predictive value was as high as 99.4% while the negative predictive value was 38.3%. They reported that the diagnosis of malignant tumors was more accurate with positive predictive value of 99.2% [5]. In our series, the only false negative case was that of osteosarcoma which was misinterpreted as osteochondroma. On review, it was found that paucicellular material on aspiration, erroneous cytological interpretation of cartilaginous components and bony trabeculae, along with lack of clinico-radiological correlation was the reason for error and such a smear should have been ideally kept under "inadequate\insufficient" category.

**Table 5:** Accuracy rates of some previous studies.

Authors	Total no. of Cases	Overall accuracy
Hajdu & Melamed *1973*^12^	86	NA
Akerman et al 1976^13^	150	80%
El Khoury et al 1983^22^	70	88%
Agarwal et al 1983^23^	69	82%
Xiaojing 1985^14^	54	76%
Layfield 1987^20^	101	87%
Kumar et al 1993^27^	79	94%
Mondal et al 1994^16^	112	96.4%
Agarwal et al 1997^5^	200	95%
Bommer et al 1997^37^	427	95%
Jorda et al 2000^2^	308	95%
Agarwal et al 2000^28^	226	86%
Wedin et al 2000^29^	110	93%
Soderlund et al 2004^11^	370	69%
Domanski et al 2005^19^	130	77%
Handa et al 2005^30^	66	93.3%
Nnodu et al 2006^32^	96	87.8%
Present series 2007	91	94.2%

We encountered 2 false positive cases. One case of chronic granulomatous bone lesion was erroneously diagnosed as metastatic adenocarcinoma with unknown primary. On review, this case showed a few large bizarre cells filled with mucin, which was subsequently found to be macrophages. Detailed clinical history, examination and radiological findings were not available at the time of FNAB. It was found that, when in doubt, adjunct stains like cytochemical and immunocytochemical markers helped in reaching a diagnosis. The other case was that of a giant cell tumor, from an elderly male with a lytic lesion of the distal humerus, which was misdiagnosed as a sarcoma (not otherwise specified). Radiological information was again not available at the time of the diagnosis. The cause of false positive diagnosis was an interpretive error where benign cells were misinterpreted as malignant. This further underlined the importance of clinico-radiological correlation in cytology. In our series, only one metastatic malignancy was found and it was correctly diagnosed. An appropriate diagnosis of a metastatic lesion by FNAB has been reported to facilitate either non-operative management as well as contemporary surgical reconstructive techniques [30].

We also analyzed the diagnostic limitations of the technique and specimen adequacy in our study group: "inadequate\inconclusive" smears were 10.9%. Most of these cases were osteosclerotic and fibro-osseous lesions due to frequent dry taps and inconclusive smears. FNAB has a limited role in diagnosing these lesions. However, an experienced aspirator (preferably the cytopathologist, as in our series), correct aspiration technique and proper radiological evaluation to locate the most appropriate site for adequate sampling may minimize chances of inadequate material being aspirated. This failure rate was consistent with rates of 1.4 – 33% reported by previous investigators [30-32].

The separation of low-grade chondrosarcoma from enchondroma (chondroma) is an important issue but since there were only 2 cases in this series, this issue could not be addressed. Layfield et al have divided chondrosarcoma into three grades depending on the proportion of chondroid and myxoid substance as well as the degree of anaplasia [6]. Similarly, Rinas et al, in a recent report, have elucidated the difficulties in sampling errors and diagnosis of dedifferentiated chondrosarcoma [33].

In recent years, cytogenetics has been helping investigators to understand the genesis of the various bone lesions. This field is still in its infancy but cytopathologists, the world over, now recognize the fact that presence of certain chromosomal aberrations worsens the overall prognosis and survival post- therapeutic intervention in few bone tumors. For example, gain of chromosome 8q23 and CDK4 alone or together with MDM2 is associated with poor prognosis in osteosarcoma [34]. Similarly rearrangement in band 8q21 is detected exclusively in aggressive chondroblastoma [35]. More and more such associations are being discovered daily in research labs world over. However, cytogenetics was not applied in the cases under the present study but their importance, as an additional investigative tool in future cannot be understated and a future study is planned to explore its relevance.

The risks of open biopsy include infection, bleeding (especially in metastases from renal carcinoma), weakening of the bone possibly leading to pathological fractures, contamination of surrounding soft tissues as well as trauma and anxiety associated with surgery. These disadvantages can be avoided if FNAB is performed as an initial investigation. Ruhs et al found FNAB to be more cost-effective than open biopsy [36]. Similarly, Bommer et al, in their elegant study, have demonstrated that initiating the investigations of bony lesion with FNAB results in considerable savings. The cost of an open biopsy in the USA has been estimated to be $US5300 as compared with $US1600 for FNAB, if both are carried out as outpatient procedures. In this day of increasing health care costs, this more than three fold cost difference itself can be an important consideration [37].

## Conclusion

FNAB is a simple and economical technique that can be performed as an outpatient procedure, reducing patient hospitalization and lowering the overall cost of patient care. Complications are few and multiple specimens can be obtained without increased morbidity. Treatment with radiation and/or chemotherapy can be initiated without any delay. In current orthopedic oncology practice, surgeons need to know the type of malignancy present. Operative approaches as well as the use of preoperative chemotherapy and radiation therapy depend on the type of malignancy diagnosed.

When sampling is adequate and radiological findings are available, FNAB of bone is a highly accurate diagnostic technique. Inflammatory conditions, benign non-fibrotic bone lesions as well as primary and metastatic malignant tumors can be correctly diagnosed. If bony lesions appears to be fibrotic and difficult to needle with the FNAB technique, a core or open biopsy may be performed. A definitive pathologic interpretation should never be rendered if diagnostic material is inadequate or radiologic information is not compatible. Therefore, radiologists, cytopathologists, and orthopedic surgeons must work together for optimal results to avoid unsatisfactory smears. We conclude that considering the overall advantages and cost-analysis, FNAB may be suggested as the initial method of choice for evaluation of bony lesions in most clinical settings, especially in resource challenged countries. The final choice should, however, be decided on the basis of the working clinical diagnosis and the institutional/personal experience.

## Competing interests

The author(s) declare that they have no competing interests.

## Authors' contributions

RM conceived the study, analysis and interpretation of data and helped to draft the manuscript.

PAS conceived the study, interpretation of data and helped to draft the manuscript.

MS participated in analysis and interpretation of data.

RM participated in the analysis of data and helped to write the manuscript

VO participated in the acquisition, analysis and interpretation of data.

PS participated in the acquisition, analysis and interpretation of data.

All authors read and approved the final manuscript.

## Supplementary Material

Additional File 1 Summary of cytological and radiological findings in bone lesions. Cytological and radiological findings that assist in diagnosis of the various bone lesions. Click here for file
