# Supplementary material for: Should fine needle aspiration biopsy be the first pathological investigation in the diagnosis of a bone lesion? An algorithmic approach with review of literature
Source: Cytojournal. 2007 Apr 17;4:9. doi: 10.1186/1742-6413-4-9 (PMC1872031; doi:10.1186/1742-6413-4-9)
Supplement: Additional File 1 — Summary of cytological and radiological findings in bone lesions. Cytological and radiological findings that assist in diagnosis of the various bone lesions. [file 1742-6413-4-9-S1.doc]

Summary of cytological and radiological findings in bone lesions.

| Lesion | Cytology | Radiology |
| --- | --- | --- |
| Osteoid Osteoma | •Sparse population of osteoblasts with  sprinkling of fibrocytes and osteoclasts.  • Osteoblasts show none of the changes of  osteosarcoma, which is an important  negative finding. | Dense cortical sclerosis with surrounding  radiolucent nidus. If centre is also ossified then  appears “target like”. |
| Osteoblastoma | • Osteoblasts like cells, singly or in groups  or in rows.  • Multi nucleated osteoclast like cells.  • Cluster of spindle cells. | A lytic well circumscribed oval or round  defect confined by a periosteal shell of  reactive bone. |
| Osteosarcoma | • Pleomorphic spindle and rounded cells.  • Tumor cells resembling Osteoblasts.  • Multinucleated tumor cells and  atypical mitotic figures.  • Presence of Osteoid (clumps of  amorphous eosinophilic material) | In most cases, mixed lytic/blastic lesion.  The radio density is “cumulus cloud like”  with tumor invasion into soft tissue. Osteoblastic  variety presents with sunburst configuration due  to periosteal reaction or “Codman’s  triangle” due to periosteal elevation.  CT/MRI helpful in delineating the extent of  tumor and Tm99 scan for predicting “skip  metastasis” and multicentricity. |
| Parosteal  osteosarcoma | • All the above features  •   Different in location | Mineralized mass attached to the cortex  With a broad base. CT/MRI useful  in evaluating the extent of medullary  involvement. |
| Chondroma | • Predominantly cartilaginous tissue  fragments.  • Cells in lacunar spaces within fragments.  • Abundant chondromyxoid ground substance. | Well- marginated tumors from radiolucent  to heavily mineralized. Mineralized pattern  is punctuate, flocculent or has ring and arc  pattern. |
| Osteochondroma |  | Character Characteristic feature is a  Projection of the cortex in continuity  with the underlying bone. Irregular  calcification is seen. Irregular  calcification is seen. A flocculent  calcification raises the suspicion of  malignant transformation. CT/MRI- continuity of  marrow spaces into the lesion. |
| Chondroblastoma | • Fragments of chondroid matrix  • Multi nucleated osteoclast-like cells.  •3 Mononuclear, rounded cells with distinct  cell borders and rounded nuclei. | Typically lytic, centrally or eccentrically placed,  relatively small (3-6 cms) lesions and are  sharply demarcated with or without a thin  sclerotic border. |
| Chondromyxoid  fibroma | •   Myxoid background substance  • Chondroid fragments.  • Spindle-shaped fibroblast-like cells,  single or in clusters.  • Osteoclast-like giant cells. | Metaphyseal, eccentric area of lysis with a  sclerotic border, cortical thinning and  extension to the subchondral plate. On  occasion it has a trabeculated “soap  bubble appearance” |
| Chondrosarcoma | • Predominantly tissue fragments in low  grade, single cells may predominate  in high-grade sarcomas.  • Abundant eosinophilic, vacuolated  cytoplasm.  • Chondromyxoid material | Fusiform Fusiform expansion and  thickening of cortical bone. It  presents as radiolucency  with variably distributed punctate  or ring like opacities  (mineralization). CT helpful in  demonstrating matrix calcification. |
| Mesenchymal  Chondrosarcoma | • All the above with presence of  mesenchymal elements. | Primarily lytic and destructive with  poor margins, not significantly differing  from ordinary chondrosarcoma. Mottled  calcification is sometimes prominent.  Expansion of the bone and cortical  destruction or cortical break through  with extra-osseous extension into soft tissue is  common. |
| Osteoclastoma | • Abundant material  • Dispersed cells and cohesive cell clusters  •    A double cell population: mononuclear  spindle cells and giant cells of osteoclastic  type.  • Giant cells are attached to the  periphery of the clustered spindle cells. | An expanding and eccentric area of  lysis with a sclerotic border, cortical  thinning and extension to the subchondral  plate. On occasion it has a trabeculated  “soap bubble appearance” |
| Ewing’s sarcoma | • Dissociated cells and clusters of cohesive  cells  • Two cell types: large pale cells  with abundant vacuolated cytoplasm  and small dark cells with scanty cytoplasm  • Abundant Cytoplasmic glycogen  • Occasionally rosette-like structures | A well defined osteolytic lesion  involving the diaphysis is the most  common feature. Permeative or moth  eaten bone destruction often associated  with “onion skin” like multi layered  periosteal reaction is characteristics. |
| Lymphoma | • A monotonous population of small  lymphoid cells to varying number of  prolymphocytes.  • Characteristics coarse granular nuclear  chromatin (“grumele”); nucleoli.  • Reed-Sternberg cells, Hodgkin cells  and variable number of eosinophils,  plasma cells and histiocytes. | Quite variable X-ray findings and  somewhat non-specific. The findings can range  from extensive lytic and sclerotic lesions to the  variable sclerosis with cortex destruction.  In absence of cortical involvement the  marrow destruction may not be obvious  on plain X-ray. Bone scan and MRI  very useful in such settings. |
| Plasma cells  Myeloma | • Many plasma cells  • Single cell presentation  • Variable cell differentiation | Lesions are lytic, sharply demarcated  and are not surrounded by a sclerotic  zone. CT/MRI may discover a very subtle  small lesion not visible on plain X-ray. |
| Haemangioma | • Limited role of cytology in diagnosis  of haemangioma as blood elements fill  up the field. | Well demarcated multiple cystic defects  that frequently contain coarse trabeculations  (often resulting in “polka dot pattern) and  striations. |
| Fibrosarcoma | • Well differentiated:  Cells are fusiform with elongated nuclei,  without any of the pleomorphism evident  in poorly differentiated fibrosarcoma  • Poorly differentiated:  Fusiform cells with elongated or ovoid  Nuclei but cells with rounded, polyhedral  or stellate shapes are also seen,  together with giant tumor cells with  large or multiple nuclei | Appears on X-ray as a destructive  geographic lesion, but may have ill defined  permeative “moth eaten” appearance  with cortical destruction and frequent soft tissue  extension. |
| Chordoma | • Abundant myxoid ground substance  • Physaliphorus cells  • Clusters of epithelial like cells.  • Pleomorphic tumor cells in some tumors. | Solitary, central, lytic, destructive lesions  of the axial skeleton. Intra-tumoral  calcification is seen particular in sacral tumors. |
| Simple bone  cyst | • Scanty population of rounded or fusiform  cells similar to as in non-ossifying fibroma.  • Random osteoclasts and osteoblasts  may be seen | Metaphysio-diaphyseal lucency, extending up  to epiphyseal plate, with little or no expansion  of bone. The cortex is usually eroded and thin,  but is intact unless pathological fracture occurs. |
| Aneurysmal bone  cyst | • Smears are heavily blood stained  and contain fusiform and rounded cells  similar to as in non-ossifying fibroma.  • Fair number of osteoclasts and  a scattering of osteoblasts | Lytic, eccentric, expansile mass with well  defined margins. Most tumors contain a thin  shell of sub-periosteal reactive bone. CT/MRI  show internal septa and characteristic fluid-fluid  level. |
| Fibrous  Dysplasia | • Sparse population of well-  differentiated fibrocytic looking cells  with mature ovoid or elongated nuclei. | Non-aggressive geographic lesion with a  ground glass matrix. There is no soft tissue  extension and a periosteal reaction is usually  not seen. |
| Langerhans Cell  Histiocytosis | •   Large histiocytes with vesicular nuclei  of irregular shape, sometimes binucleated.  • Variable number of eosinophils  • Giant cells of histiocytic type. | X-rays generally show a purely lytic, well-  demarcated lesion, usually associated with  thick periosteal new bone formation. Skull  lesions are sometimes described as “hole  in a hole”. Vertebral involvement produces  a “vertebra plana” |
